# COVID-19 and Periodontitis: A Dangerous Association?

**DOI:** 10.3389/fphar.2021.789681

**Published:** 2022-01-13

**Authors:** Francisco Javier Silvestre, Cecilia Fabiana Márquez-Arrico

**Affiliations:** ^1^ Department of Stomatology, University of Valencia, Valencia, Spain; ^2^ Department of Stomatology, University Hospital Doctor Peset-FISABIO, Valencia, Spain

**Keywords:** COVID-19, SARS-CoV-2, periodontal disease, periodontitis, periodontal infection

The COVID-19 pandemic has presented a great challenge to society, especially in the field of healthcare. An important objective was to be able to discover the new coronavirus (SARS-CoV-2) and to know the evolution and repercussions of the disease (COVID-19), as well as to identify the factors that can alter the course and form of the disease. The development in a short time of new vaccines that favor fewer severe cases and fewer hospital admissions was also a critical need.

In December 2019, several cases of acquired pneumonia were described in a group of patients in Wuhan (Hubei, China). Since then, more than 230 million people have been infected in the world and 4 million have died (World Health Organization, 2021).

COVID-19 usually occurs in a mild or asymptomatic form, but the most serious clinical form occurs in older patients and those with certain comorbidities or an acute respiratory condition that can cause respiratory distress. In these severe forms, a systemic hyperinflammatory response can occur with an exaggerated release of cytokines, which can progress to sepsis, septic shock, multi-organ failure, and death. Adults over 65 years of age have accounted for 80% of hospitalized patients, with a higher risk of complications than in younger patients. In addition, it has been associated with certain chronic diseases such as hypertension, diabetes, obesity, cardiovascular diseases, and other diseases with poor immune function such as cancer (Larvin et al., 2020; Aquino-Martinez and Hernández-Vigueras, 2021).

On the other hand, periodontitis also increases with age, is highly prevalent, and has also been related to chronic systemic diseases such as those of a cardiovascular nature, diabetes mellitus, or obesity.

Therefore, the hypothesis of the possible association between COVID-19 and periodontitis was considered. Recently, an increased risk of complications in the evolution of SARS-CoV-2 infection has been observed in patients with previous periodontitis (Borges et al., 2020; Gupta and Sahni, 2020; Larvin et al., 2020; Aquino-Martinez and Hernández-Vigueras, 2021; Campisi et al., 2021; Marouf et al., 2021).

Moreover, in the oral cavity, the epithelial cells of the oral mucosa show a high level of expression of angiotensin-converting enzyme 2 (ACE2) receptors, as well as in the fibroblasts of the periodontal ligament, and these receptors are the main entry point of the virus into the host cells. So it has been speculated that it could be a route of colonization and infection in the body. Coexpression of ACE2 receptors and the transmembrane protease enzyme SS2 (TMPRSS2), which allow the virus to enter the host cells, has been observed in these patients. TMPRSS2 and furin can cleave furin protein S of the virus. This protein also binds to CD147 to infect host cells such as the epithelial cells of the oral mucosa and gingiva. A high expression of CD147 has been observed in the gingiva and oral mucosa. The presence of SARS-CoV-2 ARS has recently been demonstrated in the crevicular fluid (GFC) (Gupta and Sahni, 2020).

In addition, periodontitis can cause changes in the structure of the periodontium due to the inflammation maintained over time in the periodontal ligament, the increase in metalloproteases that destroy the dental support tissue, and the increase in inflammatory cytokines; interleukins (ILs) 1β, 4, 6, 7, and 17; tumor necrosis factor alpha (TNF-α); and C-reactive protein (CRP). Blood parameters relevant to the course of COVID-19 such as concentrations of D-dimer, HbA1c, D vitamin, white blood cells, and lymphocytes have been found to be elevated in patients with moderate-advanced periodontitis (Marouf et al., 2021). In this way, patients with previous periodontitis could be more susceptible to exacerbations of COVID-19 (Marouf et al., 2021) (Figure 1).

**FIGURE 1:**
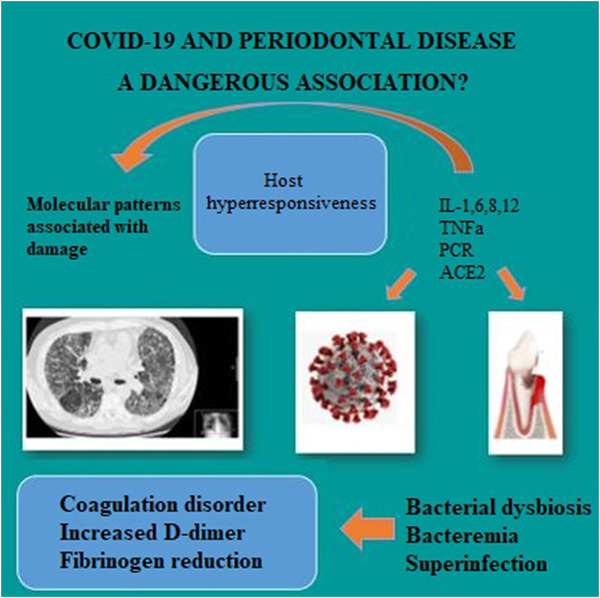
Summary of the possible routes of association between COVID-19 and periodontal disease.

Interestingly, an increase in cytokines in blood samples from patients with moderate or severe COVID-19 has been observed, and this increase in cytokines may be related to a worsening in the evolution of the disease (Marouf et al., 2021). An increase in blood cytokines has also been detected in patients with chronic periodontitis, and it has been proven that periodontal treatment favors serum reduction of the cytokine levels (Marouf et al., 2021).

Periodontal inflammation against the background of periodontitis has an overexpression of IL-6 and IL-17. IL-6 is also overexpressed alongside IL-1 when SARS-CoV-2 infects the airway. Over the course of COVID-19, the lethality of interstitial pneumonia has been linked to overproduction of IL-6 and other ILs. Therefore, there could be a link between the low-grade inflammation of advanced periodontitis and the aggravation of COVID-19 in the context of other chronic diseases (diabetes and obesity) (Campisi et al., 2021). Another mechanism of periodontitis is the so-called extracellular neutrophil entrapment (NETs), which represents an alternative form of cell death and tissue damage through immune mechanisms (Magán-Fernández et al., 2019; Borges et al., 2020; Gupta and Sahni, 2020; Magán-Fernández et al., 2020). Cell death by NETs can also be stimulated by SARS-CoV-2 (Gupta and Sahni, 2020). Exacerbation of NET production has been observed in advanced stages of COVID-19 and periodontitis, which could hypothesize a certain relationship between the physiopathogenic mechanisms of immune hyperresponse in both diseases (Magán-Fernández et al., 2019; Borges et al., 2020; Gupta and Sahni, 2020; Magán-Fernández et al., 2020). However, the mechanisms that comprise this association are yet to be determined.

Studies have recently been published where periodontitis has been linked to an increased risk of complications from COVID-19 (Magán-Fernández et al., 2019; Borges et al., 2020; Gupta and Sahni, 2020; Magán-Fernández et al., 2020; Takahashi et al., 2020; Anand et al., 2021; Campisi et al., 2021; Gupta et al., 2021; Larvin et al., 2021).

It has been speculated that the possible dissemination of periodontopathogenic bacteria inside the lower respiratory tract creates favorable conditions for suffering from the most severe SARS-CoV-2 infection through tissue damage and accelerating the process of cellular senescence at this level, as well as facilitating lung infection. Also, periodontal bacteria could be introduced through endotracheal intubation to which patients with respiratory distress are subjected (Aquino-Martinez and Hernández-Vigueras, 2021).

Besides, hospitalization of patients with periodontitis generates a situation of worsening of their oral health for the following reasons: the lack of oral hygiene in the hospitalized patients generating an increase in bacterial plaque due to the lack of good chemical–mechanical control, intubation of the patients and consequent parenteral feeding, general lack of stimulation of the salivary glands, dry mouth, and alterations in the bacterial flora (Magán-Fernández et al., 2019; Borges et al., 2020; Gupta and Sahni, 2020; Larvin et al., 2020; Magán-Fernández et al., 2020; Aquino-Martinez and Hernández-Vigueras, 2021; Campisi et al., 2021; Gupta et al., 2021; Marouf et al., 2021).

As a conclusion, we can say that COVID-19 has been recently associated with periodontitis in various studies (Magán-Fernández et al., 2019; Borges et al., 2020; Gupta and Sahni, 2020; Larvin et al., 2020; Magán-Fernández et al., 2020; Aquino-Martinez and Hernández-Vigueras, 2021; Campisi et al., 2021; Gupta et al., 2021; Marouf et al., 2021; World Health Organization, 2021). Among the mechanisms of association between both diseases, we found the inflammatory and infectious nature of both diseases shares the pull of cytokines, ILs, increased CRP, and TNF-α, among others. On the other hand, the entry pathway through the epithelia that expose a higher level of ACE2 receptors in patients with advanced periodontitis, and the increase in D-dimer present in patients with advanced periodontitis could be ways of association between both diseases. In the same way, the increase in ACE2 in periodontitis could be a reason for exacerbation of COVID-19.

## References

[B1] Anand P. S. Jadhav P. Kamath K. P. Kumar S. R. Vijayalaxmi S. Anil S. (2021). A Case-Control Study on the Association between Periodontitis and Coronavirus Disease (COVID-19). J. Periodontol. 1, 1–7. 10.1002/JPER.21-0272 34347879

[B2] Aquino-Martinez R. Hernández-Vigueras S. (2021). Severe COVID-19 Lung Infection in Older People and Periodontitis. Jcm 10 (2), 279. 10.3390/jcm10020279 PMC7828740 33466585

[B3] Borges L. Pithon-Curi T. C. Curi R. Hatanaka E. (2020). COVID-19 and Neutrophils: The Relationship between Hyperinflammation and Neutrophil Extracellular Traps. Mediators Inflamm. 2020, 8829674. 10.1155/2020/8829674 33343232 PMC7732408

[B4] Campisi G. Bizzoca M. E. Lo Muzio L. (2021). COVID-19 and Periodontitis: Reflecting on a Possible Association. Head Face Med. 17 (1), 16. 10.1186/s13005-021-00267-1 33975613 PMC8110692

[B5] Gupta S. Mohindra R. Chauhan P. K. Singla V. Goyal K. Sahni V. (2021). SARS-CoV-2 Detection in Gingival Crevicular Fluid. J. Dent. Res. 100 (2), 187–193. 10.1177/0022034520970536 33138663 PMC7642823

[B6] Gupta S. Sahni V. (2020). The Intriguing Commonality of NETosis between COVID-19 & Periodontal Disease. Med. Hypotheses 144, 109968. 10.1016/j.mehy.2020.109968 32534340 PMC7276117

[B7] Larvin H. Wilmott S. Wu J. Kang J. (2020). The Impact of Periodontal Disease on Hospital Admission and Mortality during COVID-19 Pandemic. Front. Med. (Lausanne) 7, 604980. 10.3389/fmed.2020.604980 33330570 PMC7719810

[B8] Larvin H. Wilmott S. Kang J. Aggarwal V. R. Pavitt S. Wu J. (2021). Additive Effect of Periodontal Disease and Obesity on COVID-19 Outcomes. J. Dent. Res. 100, 1228–1235. 10.1177/00220345211029638 34271846 PMC8461046

[B9] Magán-Fernández A. O'Valle F. Abadía-Molina F. Muñoz R. Puga-Guil P. Mesa F. (2019). Characterization and Comparison of Neutrophil Extracellular Traps in Gingival Samples of Periodontitis and Gingivitis: A Pilot Study. J. Periodontal Res. 54 (3), 218–224. 10.1111/jre.12621 30298590

[B10] Magán-Fernández A. Rasheed Al-Bakri S. M. O'Valle F. Benavides-Reyes C. Abadía-Molina F. Mesa F. (2020). Neutrophil Extracellular Traps in Periodontitis. Cells 9 (6), 1494. 10.3390/cells9061494 PMC7349145 32575367

[B11] Marouf N. Cai W. Said K. N. Daas H. Diab H. Chinta V. R. (2021). Association between Periodontitis and Severity of COVID-19 Infection: A Case-Control Study. J. Clin. Periodontol. 48 (4), 483–491. 10.1111/jcpe.13435 33527378 PMC8014679

[B12] Takahashi Y. Watanabe N. Kamio N. Kobayashi R. Iinuma T. Imai K. (2020). Aspiration of Periodontopathic Bacteria Due to Poor Oral hygiene Potentially Contributes to the Aggravation of COVID-19. J. Oral Sci. 23 (631), 1–3. 10.2334/josnusd.20-0388 33177276

[B13] World Health Organization (2021). WHO Coronavirus (COVID-19) Dashboard. https://covid19.who.int/ (Accessed Sep 22, 2020).

